# A Plasmid-Encoded Surface Polysaccharide Partly Blocks *Ceduovirus* Infection in Lactococci

**DOI:** 10.3390/ijms26062508

**Published:** 2025-03-11

**Authors:** Claudia Rendueles, Javier Nicolás Garay-Novillo, Martin Holm Rau, Paula Gaspar, José Ángel Ruiz-Masó, Jennifer Mahony, Ana Rodríguez, José Luis Barra, Gloria del Solar, Beatriz Martínez

**Affiliations:** 1 Instituto de Productos Lacteos de Asturias (IPLA), CSIC, C/Francisco Pintado Fe, 26, 33011 Oviedo, Asturias, Spain; claudia.rendueles@ipla.csic.es (C.R.);; 2 Departamento de Quimica Biologica Ranwel Caputto, CIQUIBIC-CONICET, Facultad de Ciencias Quimicas, Universidad Nacional de Cordoba, Cordoba X5000HUA, Argentina; jgaraynovillo@unc.edu.ar (J.N.G.-N.); jose.luis.barra@unc.edu.ar (J.L.B.); 3 R&D, Microbe and Culture Research, Novonesis A/S, 2970 Hørsholm, Denmark; marra@novonesis.com (M.H.R.); paga@novonesis.com (P.G.); 4 Centro de Investigaciones Biológicas Margarita Salas (CIB), CSIC, c/ Ramiro de Maetzu, 9, 28040 Madrid, Spain; jaruizmaso@cib.csic.es (J.Á.R.-M.); gdelsolar@cib.csic.es (G.d.S.); 5 School of Microbiology, University College Cork, T12 K8AF Cork, Ireland; j.mahony@ucc.ie; 6 APC Microbiome Ireland, University College Cork, T12 YT20 Cork, Ireland

**Keywords:** CRISPR-Cas9, dairy starters, endogenous plasmids, nanopore sequencing, phage adsorption

## Abstract

Bacteriophages (or phages) remain the leading cause of failure in dairy fermentations. Thereby, phage-resistant *Lactococcus lactis* and *Lactococcus cremoris* dairy starters are in continuous demand. In this work, our goal was to identify phage defense mechanisms against ceduoviruses encoded by two wild isolates of dairy origin named *L. lactis* IPLA517 and IPLA1064. These strains were previously subjected to experimental evolution to select derivatives that are resistant to the bacteriocin Lcn972. It was observed that the Lcn972^R^ derivatives became sensitive to phage infection; however, the underlying mechanism was not defined. The long-read sequencing technologies applied in this work reveal that all of the Lcn972^R^ derivatives shared the loss of a 41 kb endogenous plasmid (p41) that harbors a putative exopolysaccharide (EPS) gene cluster with significant homology to one described in *Lactococcus garvieae*. Using a CRISPR-Cas9-based approach, p41 was selectively cured from *L. lactis* IPLA1064. Phage infection assays with three ceduoviruses demonstrated that curing p41 restored phage sensitivity at levels comparable to the Lcn972^R^-IPLA1064 derivatives. Phage adsorption to Δp41 cells was also increased, consistent with the hypothesis of EPS production hindering access to the phage receptor protein Pip. Our results reinforce the role of EPSs in protecting *Lactococcus* against phage infection, a phenomenon that is rarely reported for ceduoviruses. Moreover, the results also exemplify the likely horizontal gene transfer that can occur between *L. lactis* and *L. garvieae* in a dairy environment.

## 1. Introduction

*Lactococcus lactis* and *Lactococcus cremoris* are key microorganisms in the production of fermented dairy products as starter cultures. Their significance extends beyond lactic acid production, encompassing the synthesis of metabolites that contribute to flavor development and biopreservation, thereby improving the overall quality and safety of the final products [1]. In this context, *L. lactis* must be able to tolerate adverse environmental conditions, including temperature fluctuations, elevated concentrations of salts, and, of particular interest in this study, the presence of bacteriophages [2].

As one of the main causes of failed fermentations or low-quality products, phages infecting *L. lactis* have been extensively studied. Lactococcal phages that are frequently associated with fermentation failures belong to *Skunavirus* (previously described as the 936 group), *Ceduovirus* (formerly known as the c2 group), and the P335 phages. [3,4]. The bacterial anti-phage arsenal, defined as genes or gene clusters that confer full or partial resistance to phage infection in the host bacterium, is of significant importance. These defense systems can be encoded in the chromosome or in mobile genetic elements and, in some cases, a distinction is made between active and passive resistance mechanisms [5]. Traditionally, numerous phage defense systems have been described in *Lactococcus*, with abortive infection (Abi) systems being the most ubiquitous. However, others such as restriction modification (R/M) and superinfection exclusion (Sie) systems are also frequently found [6]. Contrary to other bacterial genera, the CRISPR-Cas system does not appear to be a major component of the anti-phage arsenal in *Lactococcus*, as only a few CRISPR-Cas systems have been described in this genus [7]. Moreover, although its mode of action remains unclear in the majority of cases, a wide range of novel phage defense systems have emerged. In *Lactococcus*, systems such as PARIS, CBASS, or Lamassu have recently been described [8]. Additionally, passive mechanisms that hinder access to phage receptors (e.g., production of exopolysaccharides), thereby reducing the phage adsorption, may also exist [5,9]. As for phage receptors, cell wall polysaccharides (CWPSs) have been identified as the receptors for skunaviruses and P335-like phages [10]. An EPS has also been shown to be the phage receptor of some P335 phages [11]. In contrast, ceduoviruses bind reversibly to an unknown saccharide motif and irreversibly to the phage infection protein (Pip) or Pip-like proteins such as YjaE [12].

In previous adaptive evolution experiments carried out in our laboratory, in which *L. lactis* was exposed to the cell-wall-active bacteriocin lactococcin Lcn972 [13], it was observed that some of the evolved derivatives that developed resistance to Lcn972 (Lcn972^R^) became sensitive to phages CHPC1130 and CHPC1183, both classified as *Ceduovirus* members. Upon analysis of their draft genomes, different mutations were found but a common pattern could not be identified that explained their phage-sensitive phenotype. Nonetheless, a single contig sequence, harboring a putative EPS biosynthetic gene cluster and flanked by insertion sequences, was present in the parental strains and was missing in the Lcn972^R^ phage-sensitive clones. It was hypothesized that plasmid-borne EPS production may be responsible for the phage-resistant phenotype observed in the parental strains based on non-targeted curing experiments [13]. However, the presence of additional mutations hindered the ability to directly correlate the presence of a particular plasmid with the resistant phenotype. Thus, our goal in the present work was to demonstrate the role of the putative plasmid in preventing phage infection. Long-read sequencing technologies led to the identification of p41, a 41 kb plasmid-encoding the EPS biosynthetic gene cluster. Selectively curing p41 restored sensitivity to ceduoviruses, confirming the role of p41 in phage resistance.

## 2. Results

### 2.1. Chromosome- and Plasmid-Encoded Phage-Resistance Mechanisms in L. lactis IPLA517 and L. lactis IPLA1064

Hybrid genome assemblies of the phage-resistant parental strains *L. lactis* IPLA517 and IPLA1064 (Table 1) were firstly conducted to determine their plasmid content and recognizable phage-resistance mechanisms. Genome sequence analysis confirmed the close relatedness of the two parental strains, with the main difference being their plasmid content. These results agree with their common origin and the plasmid extraction analysis as previously described [14].

After manual editing of the putative plasmid sequences up to seven plasmids were identified whose main features are summarized in Table 2. Both strains share the plasmids p6, p41, p45, and p66, whereas p20 was found exclusively in *L. lactis* IPLA517. Plasmid p17 from *L. lactis* IPLA1064 is identical to p15 from IPLA517 but with a 1.7 kb insertion, likely the result of a recombination event. It is noteworthy that, except for p66, all of them have highly related RepB proteins (Figure S1).

These plasmids encode important traits in the adaptation of *Lactococcus* to the dairy niches. Plasmid p66 harbors the *lac* and *opp* operons involved in the lactose utilization and oligopeptide transport, respectively, which are frequently present in the same plasmid. This plasmid also codes for the PepO and PepF peptidases. An additional peptidase (PepT) is encoded in the p15 and p17 plasmids and, as usual, the proteinase PrtP-encoding gene is separated from the *opp* operon and found in p45. Other features commonly found in lactococcal plasmids include a complete Type I restriction/modification system in p45, as well as a single subunit of another R/M system in p6, a cadmium resistance gene in p20, along with functions involved in cell surface modifications like a putative pilus-encoding gene in p20 and an EPS gene cluster in p41 (Table 2).

According to the search with PADLOC and DefenseFinder (Table 3), the anti-phage arsenal in *L. lactis* IPLA517 and IPLA1064 is basically composed of two R/M systems and one Abi system. With the exception of the Type I R/M system, which is plasmid-encoded (p45), the remaining systems Type IV R/M and AbiH are located in the chromosome. Some other genes in the chromosome were shown to have homology to other genes that may form part of resistance systems recently described, but complete systems were not found. According to PHASTEST, both strains share one incomplete and two intact prophages in their chromosomes (Table 4), as frequently observed in strains of dairy origin [17]. Lastly, sequence comparisons of the CWPS gene cluster of both strains revealed a high identity level to that of *L. lactis* UC06 (Figure S2). The CWPSs from this strain belong to the C5 subtype whose structure is formed by repeating phospho-pentasaccharide subunits that contain arabinitol residues, rather uncommon in the lactococcal pellicle [18].

### 2.2. Loss of an EPS-Encoding 41 kb Plasmid (p41) After Experimental Evolution Under Lcn972 Pressure

Variant analyses confirmed the previously described unique pattern of mutations in the Lcn972R mutants *L. lactis* IPLA517-B5, IPLA517-C6, IPLA1064-C11, and IPLA1064-E11 (Table 1), which were predominantly found in the two-component system TCS-G, *dlt* and *pyrH* genes, among others [13]. Nonetheless, the results also reveal that all of them had lost the plasmid p41, while the plasmid p20, exclusively present in *L. lactis* IPLA517, was absent in both of the Lcn972R mutants IPLA517-B5 and IPLA517-C6. No alteration of the prophage content or the phage-resistance systems found in the parental strains was identified, pointing to the loss of p41 as the most plausible explanation for their phage susceptible phenotype.

**Figure 1:**
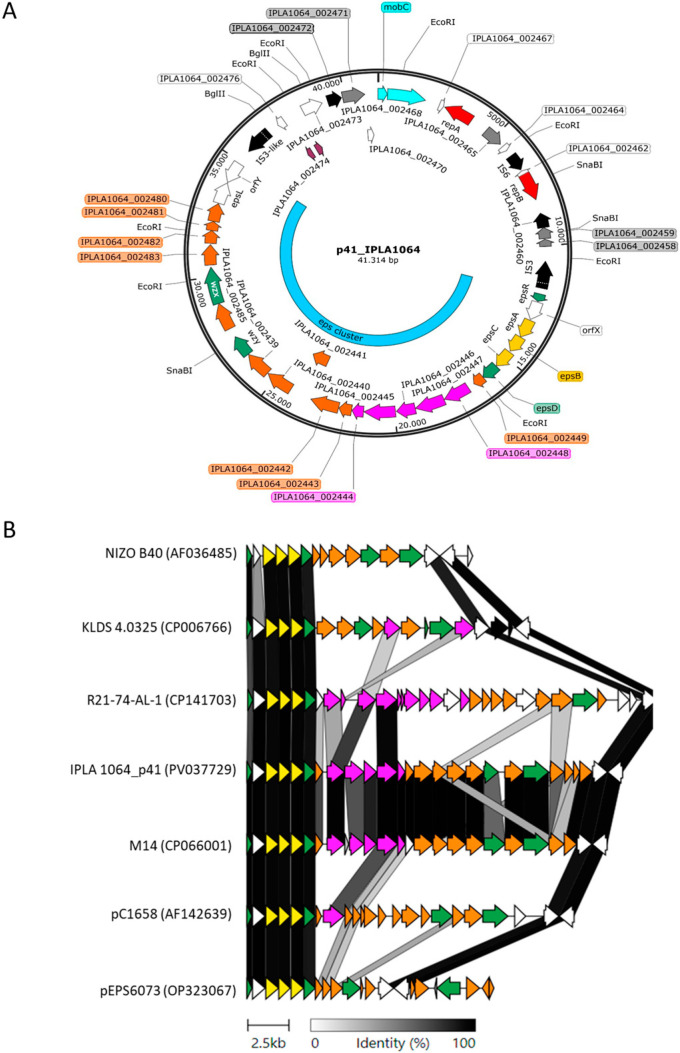
EPS biosynthetic cluster carried by plasmid p41. (**A**) p41 Plasmid map. Color code for genes (arrows): mobilization (light blue); replication (red); insertion sequences/recombinases (black); hypothetical/unknown function (white); miscellaneous (grey); EPS assembly (green); EPS modulation (yellow); EPS glycosyl transferases/sugar modification (orange); and nucleotide sugar biosynthesis (pink). The figure was created with SnapGene (https://www.snapgene.com). (**B**) Sequence relatedness of lactococcal EPS biosynthetic gene clusters. Strain codes or plasmid names are shown to the left (GenBank accession number), from top to bottom: *L. cremorisi*, B40; *L. lactis*, KLDS 4.0325; *L. petauri*, R21-74-AL-1; *L. lactis*, IPLA1064; *L. garviae*, M14; *L. cremoris*, HO2; *L. cremoris*, DGCC6073. The EPS cluster from KLDS 4.0325 is chromosome-borne. Figure was created with Clinker (https://cagecat.bioinformatics.nl/tools/clinker).

p41 is a 41.3 kb plasmid with two distinct domains (Figure 1A). One harbors the replication functions (*repB* and *repA*) and mobilization genes (*mobC*), while the other contains a 22.9 kb putative EPS biosynthetic gene cluster flanked by insertion sequences. BLASTN analyses of this putative cluster revealed a high degree of identity (99%) with a similar cluster present in a plasmid from *Lactococcus garvieae* M14 (GenBank accession no. CCXC01000006.1), isolated from Algerian fermented milk, and a certain degree of identity (40–90%) within specific genes from other *Lactococcus eps* clusters, as shown in Figure 1B. Among them, *L. lactis* and *L. cremoris* are the most represented species, followed by *L. garvieae* and *L. petauri.* Although some are chromosomally encoded, most of the *eps* clusters with identity to the one in p41 are located within relatively large plasmids, ranging in size from 20 to 63 kb. The *eps* clusters exhibit extensive variability in terms of their size and gene content with both the 5’ and 3’ ends highly conserved, while the region likely determining the EPS decoration is unique (Figure 1B). All CDSs have the same orientation, except for the last *orfY* in the 3’ end. The 5’ conserved region is composed of six CDSs with regulatory and chain-length determination functions, while the 3’ region has another two CDSs of unknown function. The variable region of the p41-*eps* cluster consists of eighteen CDSs, most of which encode putative glycosyltransferases, acetyltransferases, and enzymes likely involved in sugar-nucleotide biosynthesis. Genes predicted to encode elongation and transport functions suggest that this EPS would be synthesized via the Wzx/Wzy pathway (Figure 1).

### 2.3. Selective Curing of p41 Restores Phage Susceptibility

In order to demonstrate the role of the p41 plasmid in phage resistance, attempts to transfer the plasmid into phage-susceptible hosts were initially made. However, although the plasmid seemed to be easily mobilizable by conjugation, it was not stable in the plasmid-free *L. cremoris* MG1614 and *L. lactis* IL1403 used as receptors, and growing for a few generations resulted in the loss of the plasmid. Therefore, we opted to selectively cure p41 from *L. lactis* IPLA1064 using a CRISPR-Cas approach. This strain was chosen because of the lower number of plasmids compared to IPLA517 (Table 2).

**Figure 2:**
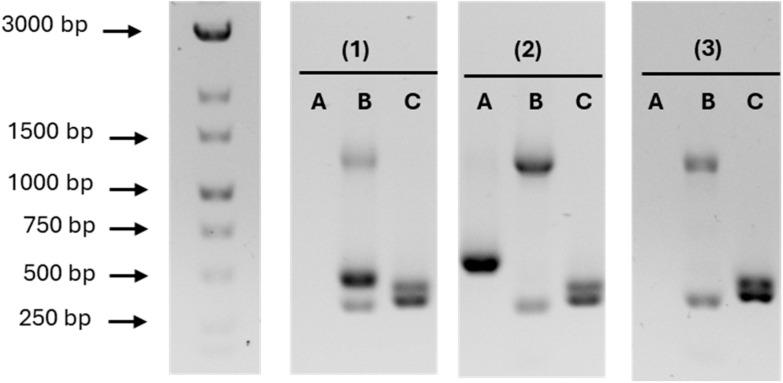
Plasmid-specific PCR assays conducted during the curing of p41. *L. lactis* IPLA1064 (**1**), *L. lactis* IPLA1064-∆p41/pILCp41LC9 (**2**), and *L. lactis* IPLA1064-∆p41 (**3**). Lane **A**: amplicon of pILCp41LC9. Lane **B** (from **top** to **bottom**) amplicons of plasmids p17, p41, and p6. Lane **C**: amplicons of plasmids p66 (**top**) and p45 (**bottom**). FastGene 1 kb DNA Marker Plus (Nippon Genetics Europe) was used as the DNA ladder.

A p41-specific 20bp-sgRNA plasmid (pILCp41LC9) was generated in *E. coli* and introduced into *L. lactis* IPLA1064 by electroporation. This plasmid targets the glycosyltransferase gene IPLA1064_002441 and contains the *cas9* gene under the control of an acid-inducible weak promoter, which was induced by growing the transformants at pH 5.5 for 45 generations, until the plasmid p41 was eliminated, as judged by plasmid-specific PCR assays (Figure 2). Subsequently, the CRISPR-Cas9 pILCp41LC9 plasmid was removed by growing the transformants at pH 7 without antibiotic selection for 150 generations. In this way, a derivative named *L. lactis* IPLA1064-Δp41, which lacks the p41 plasmid, was obtained. Notably, additional clones that had lost plasmid p41 along with another endogenous plasmid were also detected during the curing process at pH 5.5.

**Figure 3:**
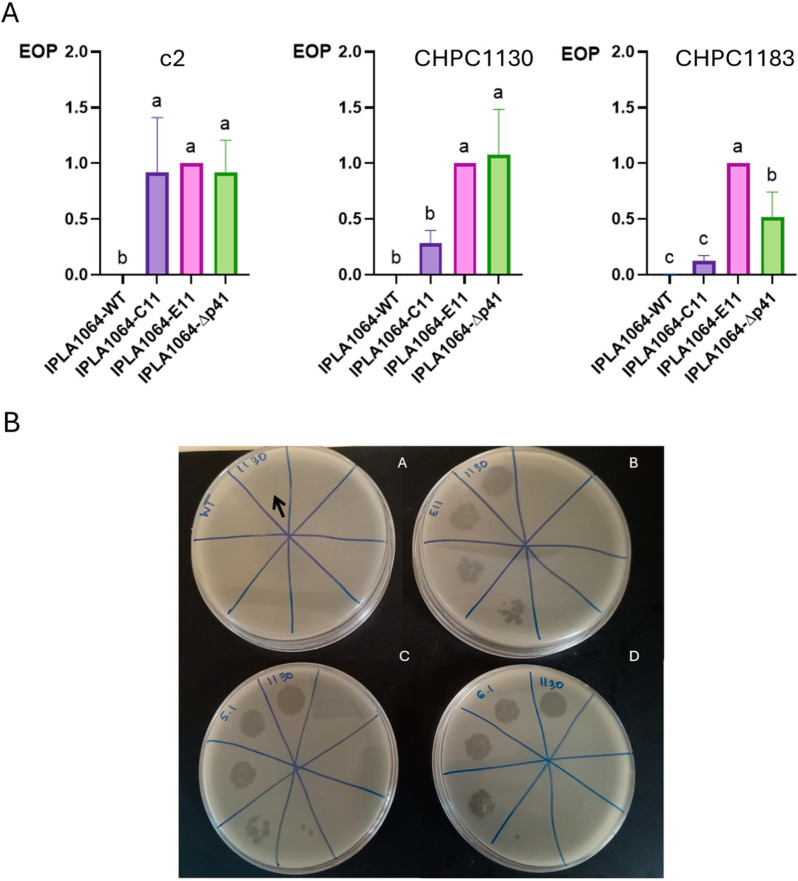
Infection of *L. lactis* IPLA1064 and derivatives by phages c2, CHPC1130, and CHPC1183. (**A**) Efficiency of plaquing (EOP) with *L. lactis* IPLA1064-E11 as a reference. The results are the mean and standard deviation of at least three independent experiments. EOPs on *L. lactis* IPLA1064 are close to 0 (0.0003 ± 0.0004 for c2; 0.001 ± 0.0004 for CHPC1130; 0.005 ± 0.002 for CHPC1183). The results with different letters (a, b, c) represent significant differences (*p* < 0.05). (**B**) Double-layer agar spot test (phage lysate tenfold dilutions) showing CHPC1130 lysis plaques on *L. lactis* IPLA1064 (A), *L. lactis* IPLA1064-E11 (B), and two clones of *L. lactis* IPLA1064-∆p41 (C,D). Due to their small size, lysis plaques on *L. lactis* IPLA1064 are hardly visible (arrow).

The impact of losing p41 on phage infection was first screened using *L. lactis* IPLA1064 and its Lcn972R derivatives as hosts in double-layer agar assays. Apart from the phages CHPC1130 and CHPC1183 described previously [13], the phage c2 was also able to form clear lysis plaques on the Lcn972R derivatives. In terms of the efficiency of plaquing (EOP), phage c2 showed the largest difference in phage infectivity among the Lcn972R derivatives and the parental *L. lactis* IPLA1064, as the EOP of c2 on *L. lactis* IPLA1064 was 0.0003 ± 0.0004, while it was of 0.001 ± 0.0004 and 0.005 ± 0.002 for CHPC1130 and CHPC1183, respectively (Figure 3). Consistently, the Lcn972R derivatives lysed when infected in broth with c2 at different multiplicities of infection (MOIs) and the percentage of inhibition was always higher, while lysis did not occur in IPLA1064 (Figure S3).

When infectivity was tested on *L. lactis* IPLA1064-Δp41 (Figure 3A), the EOP values of phage c2 and CHPC1130 resembled those of the phage-susceptible *L. lactis* IPLA1064-E11. Similarly, the size and appearance of the lysis plaques were comparable, as illustrated in Figure 3B for the phage CHPC1130. Infectivity of CHPC1183 on *L. lactis* IPLA1064-Δp41 was not fully restored (Figure 3A), likely meaning that other mutations present in *L. lactis* IPLA1064-E11 might promote infection by this phage. Overall, these results establish a link between the presence of p41 and a hindered phage infection.

### 2.4. Phage Adsorption Is Impaired in p41-Bearing Cells

**Figure 4:**
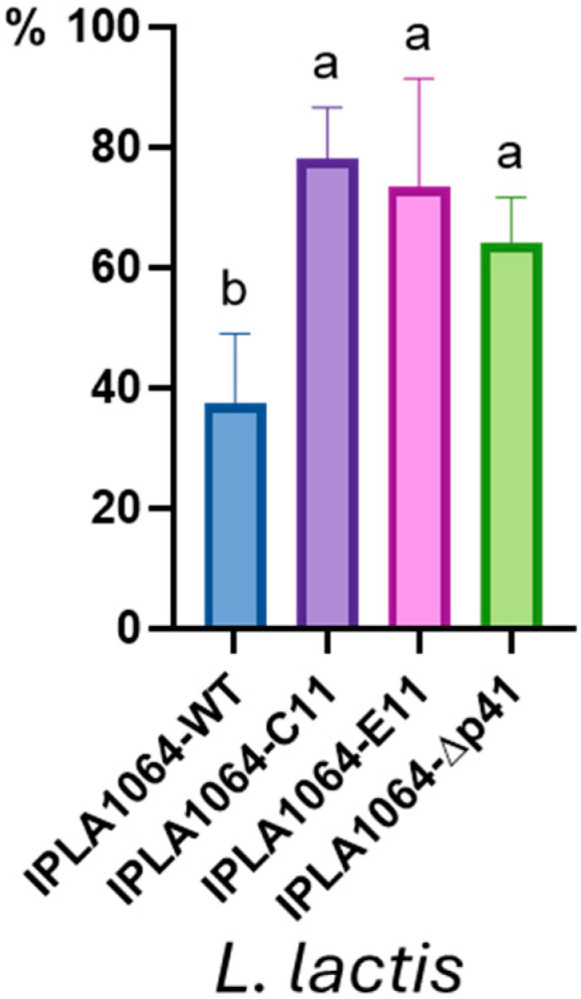
Phage c2 adsorption (%) to *Lactococcus* cells. The results are the mean and standard deviation of, at least, three independent experiments. Significant differences (*p* < 0.05) are represented by letters (a, b).

Considering that the main feature of p41 is an EPS biosynthetic gene cluster and that ceduoviruses make use of a protein receptor, we postulated that phage adsorption should be impaired in *L. lactis* IPLA1064. To test this hypothesis, phage adsorption experiments were carried out with phage c2. As shown in Figure 4, significant differences were noted within the tested lactococcal strains. The percentage of adsorbed phages to Lcn972^R^ or *L. lactis* IPLA1064-Δp41 cells approximately doubled that of the parental *L. lactis* IPLA1064, reinforcing the role of the p41-encoded EPS in hindering access to the phage receptor.

## 3. Discussion

In this work, we present evidence to establish the basis for the phage-resistance phenotype observed in *L. lactis* dairy isolates, whose Lcn972^R^-evolved clones became susceptible to c2-like phages. To this end, their genomes were scrutinized for phage-resistance mechanisms, their plasmid complement was analyzed and, finally, using a CRISPR/Cas9 approach, the phage-resistant phenotype was linked to the presence of the p41 plasmid harboring an EPS biosynthetic cluster.

Genome analyses facilitated the identification of the plasmid content of *L. lactis* IPLA517 and IPLA1064. Consistent with other dairy lactococci that often possess a large number of plasmids (up to 12), these strains harbor 6 and 5 plasmids, respectively, that encode for non-essential but beneficial features. Co-existence of plasmids with highly related RepB proteins has been previously reported in *L. lactis*, suggesting that even minor alterations in the origins of replication or the replication proteins are sufficient to prevent the incompatibility of plasmids. [19,20,21].

Regarding their anti-phage arsenal, in addition to the plasmid-encoded R/M system in plasmid p45, we found an Abi system and another R/M system in the chromosome. Incomplete R/M systems such as the one encoded by the p6 plasmid are also frequently found and may act as a complement to other complete systems [8,22,23,24,25]. However, no changes in the anti-phage systems that explain their increased sensitivity to phages were found in the Lcn972^R^-evolved clones. It is also worth noting that, apart from c2, CHPC1130 and CHPC1183, no other phages in our collection were able to infect *L. lactis* IPLA517 and IPLA1064. This could be attributed to the combination of the diverse anti-phage systems and their CWPS type, as the C5 subtype is among the less common variants [18].

Genome analyses of the Lcn972^R^-evolved clones also revealed that, as suspected, some plasmids have been lost, a phenomenon that is frequently observed under stressful conditions when the plasmids in question do not encode essential functions [26]. For example, the plasmid p41-encoding the production of a putative novel EPS had been lost in all Lcn972^R^ clones. EPS biosynthetic clusters, which are present either on plasmids or on the chromosome, are commonly found in *Lactococcus* [27]. It has been demonstrated that they may confer a selective advantage to the bacteria, providing protection against adverse conditions such as dehydration, antibiotics, toxic compounds or phages [28]. The modular organization of the EPS cluster in p41, with both 5’ and 3’ extremes conserved and all the CDSs in the same orientation except for *orfY*, is consistent with what has been previously reported [27,29]. Previous works support the idea that some EPSs may also impair phage adsorption by masking the phage receptors [9,28], pointing to the plasmid p41 behind phage resistance in the parental strain.

After the fruitless attempts at transferring the p41 plasmid to demonstrate its role in protecting against phage infection, we used a CRISPR-Cas9 system for lactic acid bacteria to selectively eliminate p41. This approach is known to be effective in the curation of lactococcal plasmids [30,31]. Elimination of p41 was observed after 45 generations at pH 5.5 in accordance with what was previously described when using the same system [16]. Curing of the CRISPR plasmid, on the contrary, was particularly challenging in this study and may be attributed to the plasmid content of this particular strain, which contains four additional plasmids. The fitness cost of maintaining an additional plasmid may not be as significant as in *L. cremoris* MG1363, a plasmid-free strain [16]. It should be noted that during this process other plasmids were also lost in addition to p41 in some of the tested clones. However, as this was only checked after the *cas9* induction, it is not possible to determine whether this loss was due to a putative lack of specificity of the system or to the instability of these plasmids at pH 5.5. The latter seems to be the most likely scenario, as previous studies have demonstrated plasmid instability during prolonged culture of lactococcal cells due to acidification [26,32]. Additionally, although it has not been tested here, it is well documented that bacterial cells with a high plasmid content tend to exhibit significant variability in plasmid numbers and, consequently, in plasmid stability. This variability can result in the emergence of diverse phenotypes when working with single colonies [19].

The selective elimination of p41 allowed us to confirm its role in *Ceduovirus* resistance because after curing p41 in *L. lactis* IPLA1064, EOP values resembled those of the Lcn972R clones. Likewise, the observation that phage adsorption to p41-bearing cells is hampered is compatible with the notion that EPS production is blocking access to the phage receptor Pip, at least partially. Consistently, loss of the ability to synthesize the EPS may also explain the increase in surface hydrophobicity previously observed in the Lcn972R derivatives [13]. To the best of our knowledge only the plasmid pCI658 has been shown to be involved in resistance to *Ceduovirus* [9], while the majority of studies have focused on the role of EPS in resistance to skunaviruses infection [9,33,34]. Prior studies with non-classified phages have also supported the idea of EPSs blocking phage receptors [28,35]. In contrast, EPSs can sometimes be necessary for infection by P335-like phages. This highlights the diverse role of EPSs in phage infection, which is dependent on both the EPSs and the phage involved [11]. Similarly, EPSs may play a dual role either as phage receptor [36] or as a phage-resistance mechanism [37] in *Streptococcus thermophilus*. In other Gram-positive bacteria, such as *Streptococcus pneumoniae* [38], *Staphylococcus aureus* [39], and *Streptococcus pyogenes* [40], capsular polysaccharides have been shown to prevent phage infection.

The observation that the EPS gene cluster in p41 is also present in *L. garvieae* is not entirely unexpected. Although *L. garvieae* is most commonly recognized as a fish pathogen and is rarely associated with human infections, dairy strains have also been described [41,42,43]. This finding reinforces the notion of horizontal gene transfer between both species, particularly when both shared a common niche, as is the case for *L. lactis* IPLA1064 and *L. garvieae* M14, which were both isolated from dairy products. While EPS biosynthetic gene clusters are also commonly found in *L. garvieae*, they have not been as thoroughly characterized as in *L. lactis* [44,45,46]. Similarly, phage resistance in *L. garvieae* has not been extensively studied. To our knowledge, only one study has linked the presence of a polysaccharide capsule to a lower infection rate by some phages [47]. Considering the similarity found among certain *L. garvieae* and other lactococcal phages infecting dairy starters, as well as the documented occurrence of cross-infection between dairy and non-dairy phages [41,48,49], it seems plausible that similar phage-resistance strategies may be employed. It would be of interest to ascertain whether the p41-encoded EPS could confer resistance to *L. garvieae* phages.

## 4. Materials and Methods

### 4.1. Bacterial Strains and Bacteriophages

Bacterial strains and phages used in this work are listed in Table 1. *L. lactis* parental strains and their Lcn972^R^ derivatives were routinely grown statically in M17 (Formedium, Hunstanton, United Kingdom) supplemented with 0.5% lactose (LM17) at 30 °C. *L. cremoris* NZ9000 was similarly grown but in M17 (Formedium) supplemented with 0.5% glucose (GM17).

Phages were propagated from previous stocks in GM17 with CaCl_2_ 10 mM (GM17*) using *L. cremoris* NZ9000 as the host strain. Phage enumeration (pfu ml^−1^) was performed by the double-layer agar assay in LM17 or GM17 with CaCl_2_ 10 mM and 0.5% glycine (LM17** or GM17**). The top agar (0.4% *w*/*v* agar) was inoculated at 4% *v*/*v* with an overnight culture of the appropriate host strain and lysate dilutions prepared in SM buffer (20 mM Tris HCl, pH 7.5, 100 mM NaCl, 10 mM Ca(NO_3_)_2_, and 10 mM MgSO_4_). The EOP was determined by dividing the titre (PFU mL^−1^) of the test strain by that of the *L. lactis* IPLA1064-E11, that was used as the reference.

### 4.2. Genome Analysis

A hybrid genome assembly approach was undertaken based on both the available Illumina short reads (Bioproject PRJNA492214) and new long-reads, created using Oxford Nanopore Technology (Oxford, UK). For long-read sequencing, genomic DNA from *L. lactis* IPLA517 and *L. lactis* IPLA1064 was extracted by genXone, S.A. (Złotniki, Poland). A total of 200 mg of the pelleted material was homogenized in a BeadBlaster™ 24 (Benchmark Scientific, Sayreville, NJ, USA). Samples were further lysed in buffer G2 from the Genomic DNA Buffer Set (QIAGEN, Hilden, Germany) with Proteinase K and RNase A, and DNA was isolated by using QIAGEN Genomic-tip 20/G kit following the Qiagen Genomic DNA Handbook. Sequencing was conducted using a GridION instrument (ONT, Oxford, UK). Illumina short reads were trimmed using AdapterRemoval v2.2.4 [50] with the following non-default parameters: “--minquality 20 --minlength 30 --trimqualities --trimns --trim5p 15”. Bacteriophage phiX DNA (sequencing control) was removed by only retrieving reads that did not map to the phiX genome, using bwa-mem2 (default parameters). No trimming or adapter removal were performed for the long reads. Hybrid genome assembly was initiated using Flye v2.9 with default parameters. (https://github.com/mikolmogorov/Flye). This was followed by one round of long-read polishing using Medaka v1.5.0 (https://github.com/nanoporetech/medaka) and two rounds of short-read polishing. First Polca [51] and masurca v4.0.8 [52] were used with default parameters, and second, polypolish v0.5.0 [53] and bwa-mem2 [54] with default parameters, in addition to the polypolish_insert_filter parameter.

Variant analysis was conducted with the breseq bacterial variant calling pipeline [55] using the default parameters. For a variant to be called, at least 80% of reads mapping to a position was required to harbor the variant. The PGAP [56] annotated hybrid assembly was applied as a reference while the corresponding short reads of each strain were used as input.

The presence of known phage-resistance systems was analyzed by using the available bioinformatical tools PADLOC and DefenseFinder [57,58]. Additionally, prophages encoded within the genome were identified using PHASTEST [59].

### 4.3. Plasmid Assemblies and Confirmatory PCRs Reactions

Putative plasmid sequences were manually edited and confirmed by PCR. Primers (Table S1) were designed considering the sequences at the beginning and the end of the putative plasmid contigs and PCR reactions were performed on total DNA in order to close and confirm their sequences. PCR reactions were performed using the 2× Taq master Mix (AMPLIQON, Odense, Denmark) following the manufacturer’s instructions. The amplified DNA fragments were purified with the PCR DNA and Gel Band Purification kit (Cytiva, Buckinghamshire, UK), and their nucleotide sequences determined by Sanger sequencing (Macrogen, Madrid, Spain). The PCR conditions were as follows for all the plasmids, with the exception of p20 where the annealing step was performed at 50 °C: an initial denaturation cycle at 98 °C for 10 s, 35 cycles of a denaturation step at 98 °C for 30 s, an annealing step at 55 °C for 30 s, an extension step at 72 °C for 1 min, and a final extension cycle at 72 °C for 10 min.

### 4.4. Selective Plasmid Curing

A CRISPR-Cas9 plasmid harboring a specific single-guide RNA (sgRNA) with a spacer for the elimination of the p41 (sgp41) plasmid was constructed. This 20 bp spacer was designed using “CCTop—CRISPR/Cas9 online predictor” (https://cctop.cos.uni-heidelberg.de/) [60] with no possible mismatches to avoid off-target occurrence in the *L. lactis* IPLA1064 chromosome or plasmids. The 5′ phosphorylated primers P41sgRNAFor and RNDcrePR1Rev (Table S1) were used to amplify and generate the vector with sgp41 sequence by an inverse PCR approach using DNA of pILCsgLC9 as template. [16]. The PCR product corresponding to the linear vector with the p41sgRNA was then subjected to intramolecular ligation, heated to 65 °C to inactivate T4 DNA ligase and digested with DpnI to degrade methylated DNA from the template. Finally, the ligation mixture was used to transform *E. coli* NZY5α (NZYTech, Lisbon, Portugal) competent cells. The pILCp41LC9 construct obtained in *E. coli* was then introduced into *L. lactis* IPLA1064-WT by electro-transformation, as previously described, using chloramphenicol (Cm) 5 µg/mL as the selective marker [61]. The expression of the *cas9* gene, located under the control of the acid-inducible weak P170-578 promoter [16], was induced by cultivation in LM17,f adjusted to pH 5.5 by adding HCl 37% (LM17-5.5). To achieve the selective elimination of p41, a single transformant colony was inoculated into liquid LM17-5.5 medium supplemented with Cm and incubated for 45 generations of serial cultivation. Five colonies obtained by plating the previous culture on LM17-5.5 agar plates containing Cm were tested by colony PCR using multiple primer pairs for parallel amplification of a target sequence from each endogenous plasmid. A colony retaining all the plasmids except p41 was selected. Next, pILCp41LC9 was cured by growing the selected clone for 150 generations in liquid LM17 medium in the absence of Cm. Three Cm-sensitive colonies were selected that did not show the pILCp41LC9 (tested with primers_Cas9ForEnd and sgp41R, Supplemental Material) but contained all the endogenous plasmids except p41 (*L. lactis* IPLA1064-Δp41). Detection of p41 and pILCp41LC9, as well as the rest of the plasmids, was performed by PCR with the appropriate primers (Table S1). In all cases, PCRs were performed using Phusion DNA Polymerase (New England Biolabs, Ipswich, MA, US). The correct nucleotide sequence of all novel constructs was confirmed by automated DNA sequencing.

### 4.5. Phage Assays

Progression of infection by the phage c2 in broth was followed by measuring the optical density (OD) at 600 nm each 15 min for 24 h at 30 °C in a microtiter plate reader (Tecan Trading AG). Each *L. lactis* strain was inoculated at 2% *v*/*v*, grown until an OD of 0.2 (approx. 5 × 10^7^ cfu mL^−1^) and subsequently diluted 1:10 or 1:100. The three cultures were mixed with the phage c2 in a 3:1 ratio in a ninety-six well-plate to achieve a multiplicity of infection (MOI) of approximately 10, 1, and 0.1. CaCl_2_ was added at a final concentration of 10 mM. The area under the curve (AUC) after 24 h of incubation and the percentage of inhibition (% PI) were determined as previously described [62].

Phage adsorption assays were performed as previously described with some modifications [63]. Each *L. lactis* culture was grown until an OD of 0.5 and mixed with the phage c2 at a MOI of 0.01 in the presence of 10 mM CaCl_2_. Samples were incubated for 10 min at 30 °C and, after centrifugation, the non-adsorbed phages (residual) were quantified. A sample without cells was equally treated to determine the initial phage titre (control). Experiments were carried out with at least three biological replicates and the percentage of adsorption was calculated as (1−residual/control) × 100.

### 4.6. Statistical Analyses

The EOP and adsorption data were subjected to statistical analysis using a one-way ANOVA, followed by a Tukey’s multiple comparisons test, employing GraphPad Prism 6 software (Boston, MA, USA). Differences were considered significant when *p* < 0.05.

## 5. Conclusions

In this work, we demonstrated that the p41 plasmid containing an EPS biosynthetic gene cluster was responsible for the *Ceduovirus* resistance in *L. lactis* IPLA1064. This EPS may block access to the phage receptor, impeding phage adsorption and reducing the efficiency of the infection. Further work could be conducted in two distinct areas in relation to this study. First, the chemical and technological characterizations of the EPS and the EPS-producing *L. lactis* strains could facilitate their potential application in the dairy industry, e.g., as a texturing agent. Conversely, it would be beneficial to ascertain if it is also implicated in phage resistance in *L. garvieae* and whether or not the plasmid p41 can be transferred to clinical or pathogenic strains, which would make the potential use of phage therapy in this field more challenging.

## Figures and Tables

**Table 1:** Bacterial strains, phages, and plasmids used in this study.

Strain	Description	Reference
*Lactococcus cremoris*		
NZ9000	*L. cremoris* MG1363 *pepN::nisRK*. Host for phage propagation	[15]
*Lactococcus lactis*		
IPLA517	Phage-resistant; 6 endogenous plasmids	[14]
IPLA517-B5	IPLA517 Lcn972^R^-evolved clone; phage-sensitive; 4 endogenous plasmids	[13]
IPLA517-C6	IPLA517 Lcn972^R^-evolved clone; phage-sensitive; 4 endogenous plasmids	[13]
IPLA1064	Phage-resistant; 5 endogenous plasmids	[14]
IPLA1064-C11	IPLA1064 Lcn972^R^-evolved clone; phage-sensitive; 4 endogenous plasmids	[13]
IPLA1064-E11	IPLA1064 Lcn972^R^-evolved clone; phage-sensitive; 4 endogenous plasmids	[13]
IPLA1064-Δp41	IPLA1064 cured of plasmid p41	This work
Bacteriophages		
c2	*Ceduovirus* (GenBank NC_001706)	Lab collection
CHPC1130	*Ceduovirus*	Novonesis collection
CHPC1183	*Ceduovirus* (GenBank MN689511)	Novonesis collection
Plasmids		
pILCsgLC9	Derived from pIL253 with a CRISPR-Cas9 cassette for specific spacers cloning	[16]
pILCp41LC9	Derived from pILCsgLC9 with a specific spacer for p41 curation	This work

**Table 2:** Size and main features of endogenous plasmids found in *L. lactis* IPLA517 and *L. lactis* IPLA1064.

Plasmid (Accession Number)	Size (bp)	Present in	Identity IPLA1064 vs. IPLA517	Main Features
p6 (PV037731)	5860	Both	100%	RepB; R/M subunit
p15 (PV037733)	15315	IPLA517	NA ^1^	RepB and RepX; MobC; Pyrimidine synthesis genes (*tmk*, *pyrF*, *cdd*)
p17 (PV037728)	16890	IPLA1064	99% to IPLA 517_p15 plus a 1678-bp insertion	RepB and RepX; MobC; Pyrimidine synthesis genes (*tmk*, *pyrF*, *cdd*)
p20 (PV037734)	20376	IPLA517	NA ^1^	RepB Pili genes; *cadA*
p41 (PV037729)	41314	Both	100%	RepB and RepA; MobC; EPS cluster
p45 (PV037730)	45496	Both	99%	RepB; MobC; Type I R/M system; PrtP and Clp (protease)
p66 (PV037732)	66477	Both	100%	RepB; MobC *lac* operon; *opp* operon; *pepF* and *pepO*

^1^ NA: not applicable.

**Table 3:** Phage-resistance systems present in both *L. lactis* IPLA517 and *L. lactis* IPLA1064 as identified through DefenseFinder and PADLOC.

Resistance System	Location (*L. lactis* IPLA1064 Coordinates)	Hit Gene	Blastp (% Identity/Query Cover)	Identified by
Restriction-modification system type IV	Chromosome (87858–89420)	RM—RM_Type_IV	WP_081213772. McrB family protein (99.81%/100%)	DefenseFinder
AbiH	Chromosome (788281–789141)	AbiH—AbiH	WP_153004554. AbiH family protein (99.65%/100%)	DefenseFinder
Restriction-modification system type I	Plasmid (p45) (7069–12972)	RM__Type_I_REases; RM__Type_I_MTases; RM__Type_I_S	WP_124137260.1. Type I restriction endonuclease subunit R (100%/100%); WP_124137261.1. Type I restriction–modification system subunit M (100%/100%); WP_124137264.1. Restriction endonuclease subunit S (100%/100%)	PADLOC and DefenseFinder

**Table 4:** Prophage content of both *L. lactis* IPLA517 and *L. lactis* IPLA1064 based on PHASTEST output.

Prophage	Region Length	Position in *L. lactis* IPLA1064	Closest Phage (%Identity/Query Cover)	Observations
#1	40 kb	178022–218024	ul36 (NC_004066) (93.27%/50%)	Intact
#2	20.4 kb	464894–485327	bIL3140 (NC_002669) (86.74%/2%)	Incomplete
#3	46.1 kb	724573–770760	PLgT-1 (NC_031016) (82.58%/33%)	Intact

## Data Availability

The original contributions presented in this study are included in the article/Supplementary Material. Further inquiries can be directed to the corresponding author. Manually curated plasmid sequences have been deposited at the NCBI, with the following accession nos: PV037728-IPLA1064_p17; PV037729-IPLA1064_p41; PV037730-IPLA1064_p45; PV037731-IPLA1064_p6; PV037732-IPLA1064_p66; PV037733-IPLA517_p15; and PV037734-IPLA517_p20. DNA sequences have been deposited in the SRA archive under the project PRJNA492214.

## Supplementary Materials

The following supporting information can be downloaded at: https://www.mdpi.com/article/10.3390/ijms26062508/s1. References [64,65,66] are cited in the Supplementary Material.

## Abbreviations

The following abbreviations are used in this manuscript:

EPS	Exopolysaccharide
Abi	Abortive infection
R/M	Restriction modification
Sie	Superinfection exclusion
CWPSs	Cell wall polysaccharides
Pip	Phage infection protein
Lcn972^R^	Lactococcin 972-resistant derivatives
EOP	Efficiency of plaquing
MOI	Multiplicity of infection
AUC	Area under the curve
PI	Percentage of inhibition
OD	Optical density
