# Supplementary material for: A Plasmid-Encoded Surface Polysaccharide Partly Blocks Ceduovirus Infection in Lactococci
Source: Int J Mol Sci. 2025 Mar 11;26(6):2508. doi: 10.3390/ijms26062508 (PMC11942015; doi:10.3390/ijms26062508)
Supplement: Supplementary file 1 [file ijms-26-02508-s001.zip › ijms-3515943-supplementary.pdf]

Table S1. Primers used in this work.

Name	Sequence	Purpose
**p6-Fw**	GGATAGCACCACTATCTAATTTTG	Detection of plasmid p6
**p6-Rv**	GGACACGGCACTTATGGC	
**p15-Fw[1]**	GATAAACCGTATTGTCTTCCAATGG	Detection of plasmids p15 and p17
**P15-Rv[1]**	GGCGAGCTTGTTGCAATAAAGAG	
**p15-FwN[1]**	CAAAGGAGAAAATGAATGATGATTAAC	
**p15-RvN[1]**	GACCGGAAACATATTCAAAGTC	
**p41-Fw**	CTCGTTCCCTCTTATACAAATAACTAAG	Detection of plasmid p41
**p41-Rv**	GTGTGGTGGAAAAACGGGGCTTG	
**p45-Fw**	CAGCCAGTACTTCTTGAGTCC	Detection of plasmid p45
**p45-Rv**	CGCAAATCAGGTCATGACAATAAAAC	
**p66-Fw**	CCTTACCAGGCCGAAGTGG	Detection of plasmid p66
**p66-Rv**	GAGCCGTATACTCCGAGAGG	
**P41sgRNAFor**	GCTTTGGGACGTATTCTGCAGTTTTAGAGCTAGAAATAGCAAGTTAAAATAAGG	iPCR for the cloning of sgp41 by introduction of p41 specific 20bp-spacer
**RNDcrePR1Rev**	TTAGTTATTATACTACTTCAAATCGATATTTGCAAGTTAC	
**Cas9ForEnd**	GCGTGTTATTTTAGCAGATGCC	Detection of plasmid pILCp41LC9
**sgp41R**	TGCAGAATACGTCCCAAAGC	

[1]p15-FwN and p15-RvN were used for Sanger sequencing of the p15 and p17 plasmids. p15-Fw and p15-Rv were employed in specific PCR reactions for detecting p15/p17.

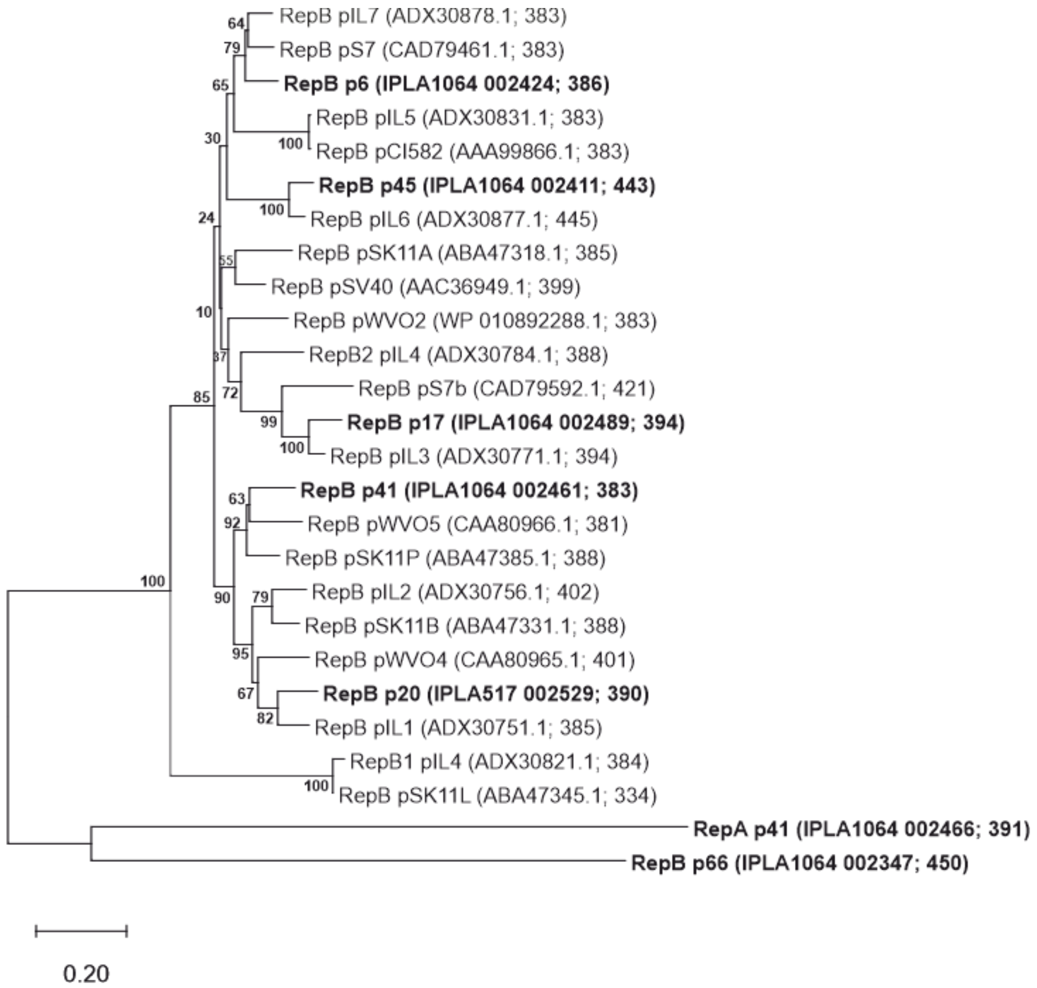

Figure S1. **Neighbor-joining phylogenetic rooted tree based on the replication protein sequences of several lactococcal theta-type plasmid replicons.** Evolutionary analyses were conducted in MEGA11 [1] The percentage of replicate trees in which the associated taxa clustered together in the bootstrap test (1000 replicates) is shown next to the branches [2]. The tree is drawn to scale, with branch lengths in the same units as the evolutionary distances used to infer the phylogenetic tree. Evolutionary distances were calculated using the Poisson correction method [3] and are in units of the number of amino acid substitutions per site. This analysis included 26 amino acid sequences of RepB proteins. For each sequence pair, all ambiguous positions were removed (pairwise deletion option). There was a total of 500 positions in the final data set. Accession numbers or identifiers for those sequences not deposited in the GeneBank are given in parentheses. RepB proteins of the plasmids reported in this study are shown in bold.

References

1. Tamura K., Stecher G., and Kumar S. (**2021**). MEGA 11: Molecular Evolutionary Genetics Analysis Version 11. *Molecular Biology and Evolution* https://doi.org/10.1093/molbev/msab120.
2. Felsenstein J. (**1985**). Confidence limits on phylogenies: An approach using the bootstrap. *Evolution* **39**:783-791.
3. Zuckerkandl E. and Pauling L. (**1965**). Evolutionary divergence and convergence in proteins. Edited in *Evolving Genes and Proteins* by V. Bryson and H.J. Vogel, pp. 97-166. Academic Press, New York.

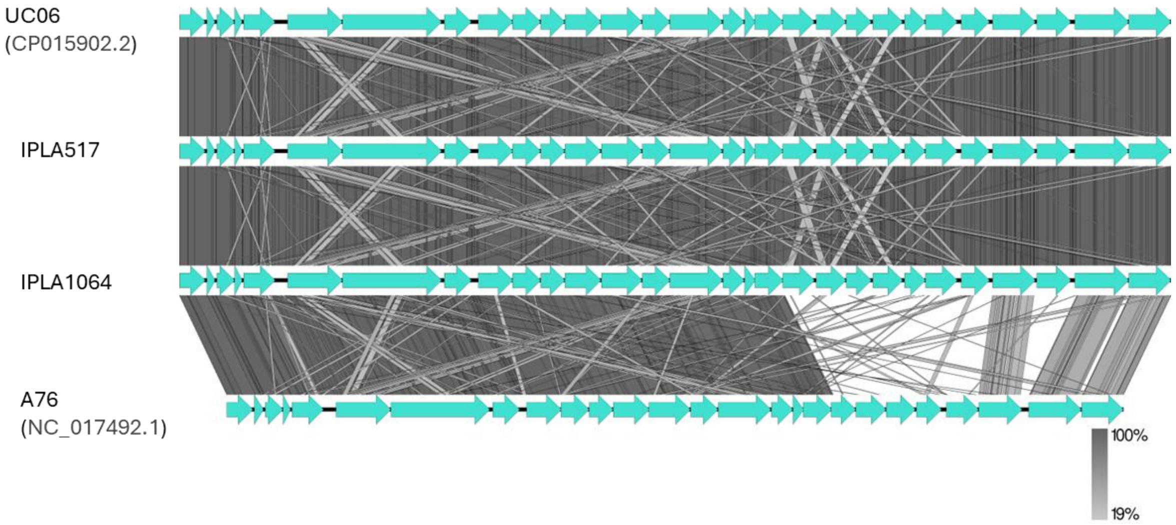

Figure S2. Sequence relatedness of *L. lactis* IPLA1064 and *L. lactis* IPLA517 CWPS biosynthetic gene clusters with the closest CWPS types (C5 and C6 type). Strain codes are shown to the left (GenBank accession number). From top to bottom: *L. lactis* UC06 (CWPS type C5); *L. lactis* IPLA517 (CWPS type C5); *L. lactis* IPLA1064 (CWPS type C5); *L. cremoris* A76 (CWPS type C6).

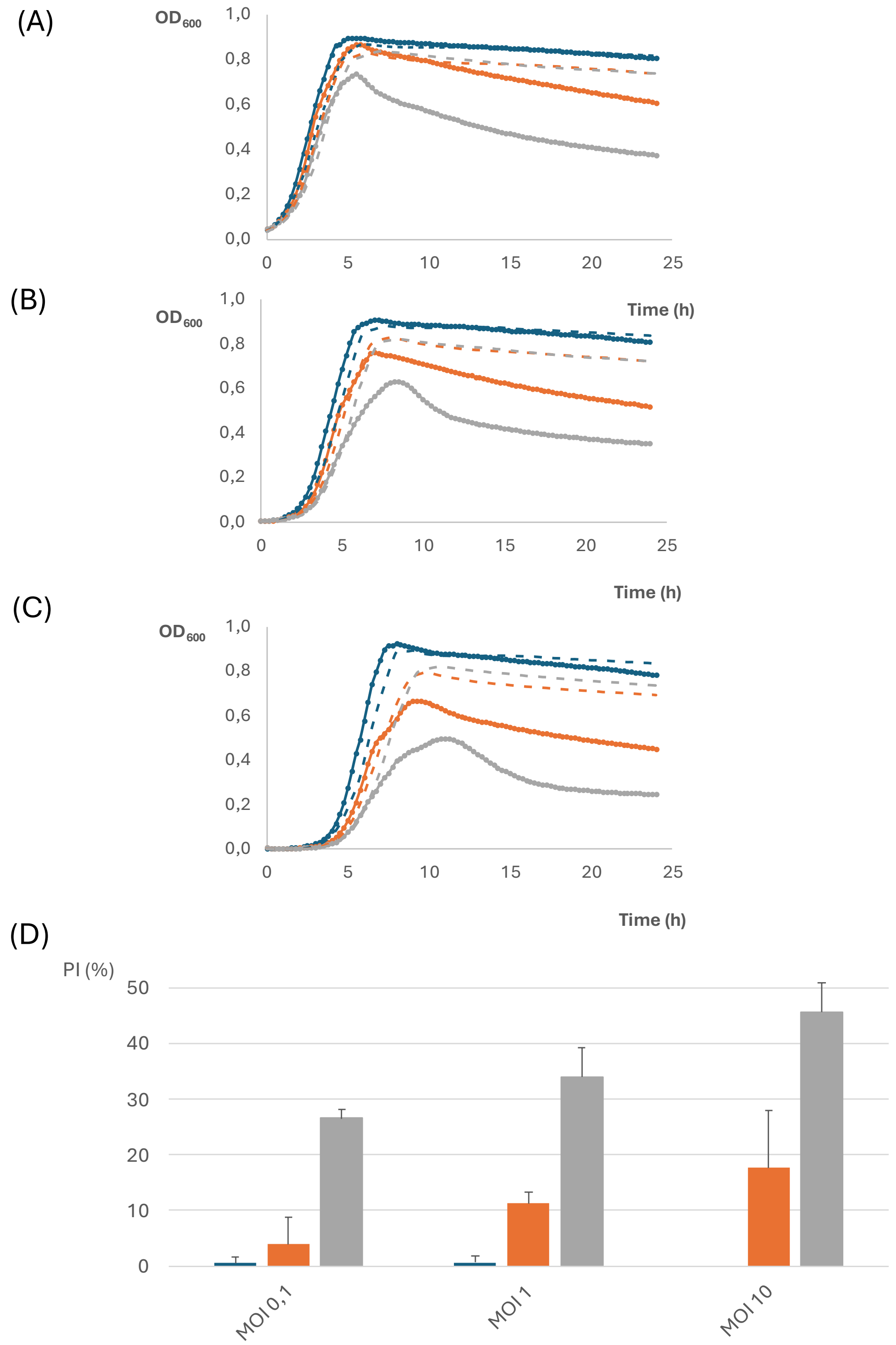

Figure S3. Phage c2 infection in broth. *L. lactis* IPLA1064 (blue), *L. lactis* IPLA1064-C11 (orange), and *L. lactis* IPLA1064-E11 (grey). Phage infection at a MOI of 10 (A), 1 (B) and 0.1 (C). Curves are presented as the mean of three replicates, and the standard deviation is not included for clarity. Both control cultures without phage (dashed lines lines) and infected cultures (solid lines) are shown. Calculated percentage of inhibition is shown in (D).
